# Coronavirus Vaccination: Spike Antibody Levels in Health Workers after Six Months—A Cross-Sectional Study

**DOI:** 10.3390/ijerph191811422

**Published:** 2022-09-10

**Authors:** Lukas Damerau, Georg Mühlenbruch, Agnes Evenschor-Ascheid, Christine Fussen, Albert Nienhaus, Claudia Terschüren, Robert Herold, Volker Harth

**Affiliations:** 1 Institute for Occupational and Maritime Medicine (ZfAM), University Medical Center Hamburg-Eppendorf (UKE), 20459 Hamburg, Germany; 2 Rhine-Maas Hospital, District Aachen, 52146 Würselen, Germany; 3 Institute for Health Services Research in Dermatology and Nursing (IVDP), Centre for Epidemiology and Health Services Research for Healthcare Professionals (CVcare), University Hospital Hamburg-Eppendorf (UKE), 20246 Hamburg, Germany; 4 Department of Occupational Medicine, Toxic Substances, Health Service Research, German Statutory Institution for Accident Insurance and Prevention for Health and Welfare Services (BGW), 22089 Hamburg, Germany

**Keywords:** COVID-19, SARS-CoV-2, occupational health, vaccine, anti-spike

## Abstract

Healthcare workers bear a high risk of infection during epidemics and pandemics such as the current SARS-CoV-2 pandemic. Various new vaccines have been approved. We investigated the influence of the time elapsed since vaccination, as well as of vaccination schema, on health workers’ spike antibody levels following their second vaccination. Blood samples were obtained from employees working at a German hospital between August 2021 and December 2021 on average half a year (range 130–280 days) after their second vaccination. Levels of SARS-CoV-2-IgG antibodies (spike and nucleocapsid protein) were qualitatively detected via chemiluminescent immunoassays (CLIAs). A previous infection with SARS-CoV-2 was an exclusion criterion. In total, 545 persons were included in this cross-sectional study. Most participants (97.8%) showed elevated anti-spike concentrations. Anti-spike levels differed significantly among vaccination schemas. Repeated vector vaccinations resulted in lower protective antibody levels. Higher age levels, immunosuppression and a longer time period since the second vaccination resulted in lower anti-spike levels. Women’s antibody levels were higher, but not significantly. Since anti-spike levels drop after vaccination, further boosters are required to increase immunoreactivity. If two vector vaccines have been administered, it is possible that an mRNA booster might increase the anti-spike level.

## 1. Introduction

Healthcare workers bear a high risk of infection during epidemics and pandemics such as the current SARS-CoV-2 pandemic [1,2,3,4,5]. This is due to direct contact with patients, which is necessary during diagnostic examinations, therapeutic and life-saving measures and nursing activities.

Vaccination is currently an important protective measure against SARS-CoV-2, and vaccines were developed rapidly in 2020 [6,7]. During 2021, citizens in Germany were vaccinated against SARS-CoV-2 according to defined prioritisation levels. In setting prioritisation, the vaccination ranking was determined according to factors including age and occupational risk of infection. Physicians and nurses in intensive care and normal wards who cared for COVID-19 patients at the beginning of the pandemic were part of the first group to receive vaccination.

Various new vaccines have been approved for immunisation in the EU and Germany. Comirnaty^®^ by BioNTech (Mainz, Germany)/Pfizer (New York, NY, USA) and Spikevax^®^ (previously named COVID-19 Vaccine Moderna^®^) by Moderna (Cambridge, MA, USA) are available as mRNA COVID-19 vaccines, for example. These are gene-based vaccines that contain the mRNA (messenger RNA or messenger ribonucleic acid) as a “blueprint” for the SARS-CoV-2 spike protein that the virus presents on its surface. No replication-capable viruses can be produced based on COVID-19 mRNA vaccines [8]. Alternatively, COVID-19 vector vaccines such as Vaxzevria^®^ by AstraZeneca (Cambridge, UK) and COVID-19^®^Vaccine Janssen by Janssen Cilag International (Beerse, Belgium)/Johnson & Johnson (New Brunswick, NJ, USA) are available. These are also gene-based vaccines that use vector viruses. The vector virus is a well-known, well-studied virus that transports the genetic information for the SARS-CoV-2 spike protein into defence cells in the body [9]. The blueprint for the protein is “read” there, and these cells then produce the spike protein. In the body of the vaccinated person, the spike proteins formed in this way are recognised by the immune system as foreign proteins. As an immune response, antibodies are formed against the SARS-CoV-2 spike protein [8].

There is debate about how long this initial immunisation lasts, and whether it offers nearly complete or a high level of vaccination protection. Initial studies show that, firstly, vaccination protection against the Delta and Omicron variants of SARS-CoV-2 may decrease [10,11]. Secondly, the effectiveness of the immunisation may diminish over a period of months, and another booster vaccination may be needed [12,13]. Antibodies against the SARS-CoV-2 spike protein aim to enable the immune system to neutralise the virus [14]. Spike IgG antibody titres also correlate with breakthrough infections and are important for protection against SARS-CoV-2 [15,16]. Previous studies have shown that the immune response can vary in strength depending on factors such as gender and age [16,17].

This study controlled for age, gender and immune status to investigate how elapsed time and the vaccine schema influence the level of protection against SARS-CoV-2. Beyond informing the respective health worker and the occupational health department about the immune protection of workers in a particular hospital, the study intends to contribute to the prospective development of vaccination strategies for the medical, nursing and other staff groups over the further course of the pandemic, and to assist in planning for and ensuring preventive infection protection for employees.

## 2. Materials and Methods

### 2.1. Recruiting

We conducted the study at the Rhein–Maas–Klinikum (RMK), one cooperation partner for the study. This primary care hospital is located in the vicinity of the Heinsberg district, one of the first hotspots in Germany, North Rhine–Westphalia, and is of particular interest because it was highly affected by the first COVID-19 wave in spring of 2020. A previous study reported a prevalence of SARS-CoV-2 of n = 52 (5.8%) at the RMK [18]. Clinic staff in all areas were vaccinated depending on priority group and vaccine availability provisioned by the federal and state ministries of health.

**Figure 1:**
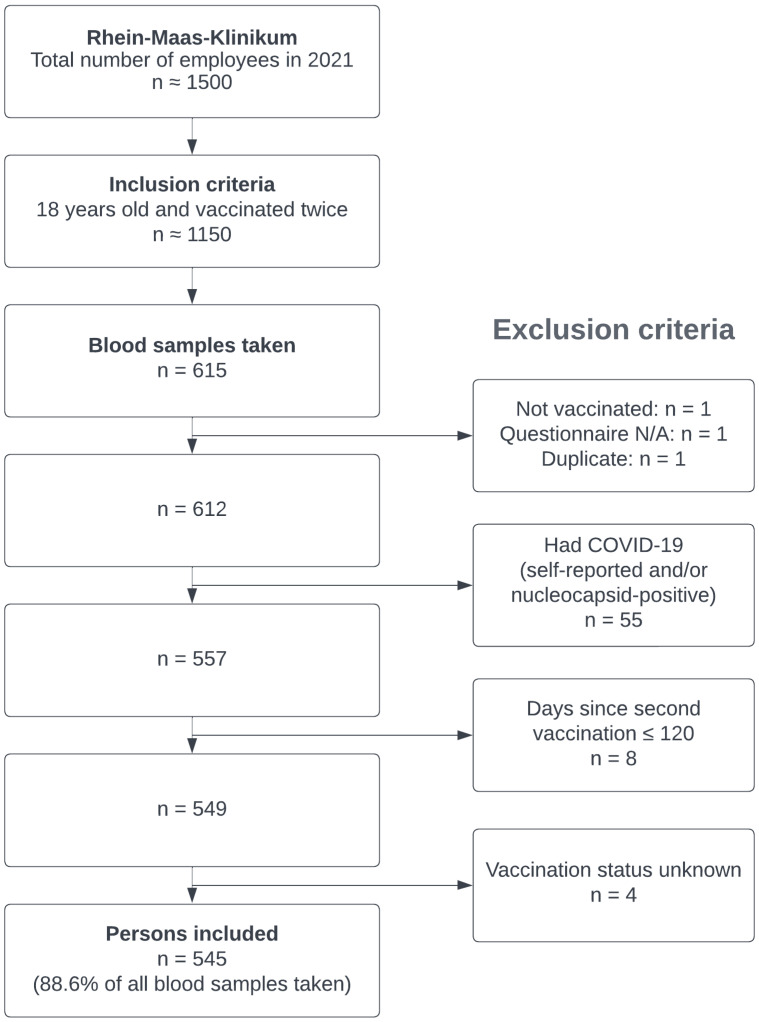
Flowchart of the recruitment and sample size.

This cross-sectional study was conducted from 1 August 2021 to 1 December 2021. All employees who were at least 18 years old and had been vaccinated twice were included. Employees were excluded if they had previously undergone a SARS-CoV-2 infection (self-reported in the questionnaire or determination of nucleocapsid antibodies), if fewer than 120 days had passed between their second vaccination and the blood collection, or if the vaccination status was not sufficiently detectable. Participation in this study was voluntary. Employees were informed about the study by email and in the news section of the intranet. There was no remuneration for participation. At the time of data collection, the primary care hospital employed around 1500 persons in total. Complete data were available for n = 545 persons (see Figure 1). Every participant consented to the study in writing. All data were pseudonymized. An association to the actual health worker was only known to the occupational health physician. This study was approved by the Hamburg Medical Association Ethics Committee (Reference 2021-100694-BO-ff). The principles of the Helsinki Declaration were followed.

### 2.2. Sample Collection/Outcome

The outcome variable was the SARS-CoV-2 anti-spike IgG concentration at least four months after the second vaccination. Blood samples (one serum monovette) were collected from all participants in the study. The RMK provided staff to coordinate and collect the blood samples and to record and code the data. The occupational health physician and her staff performed most of the blood collection. Further blood collection was carried out by trained and experienced hospital staff in specific clinical departments.

The serological examination was carried out by an external laboratory (MVZ SYNLAB Leverkusen GmbH), which has a local branch at the RMK. Some of the laboratory staff and the laboratory manager are employees of the RMK. The blood samples were qualitatively tested for SARS-CoV-2 antibodies (IgG) using a chemiluminescent immunoassay (CLIA) according to the manufacturer’s instructions (see Supplementary Materials S1). A LIAISON^®^ XL analyser was used for testing. The upper cut-off limit was 2080 binding antibody units (BAU) per mL for laboratory sensitivity reasons. The laboratory provided the threshold value above which a person is considered vaccinated. The threshold was >33.8 BAU/mL anti-spike IgG.

### 2.3. Explanatory Variables

A unified questionnaire (see Supplementary Materials S2) recorded socio-demographic data and the exposure to potential contagion at work and in private. The questionnaire also asked when the first/second vaccination was received (only month, always imputed to the 15th), which vaccine was used and if and what side effects occurred, respectively. The number of reported side effects was transformed in a variable with six levels (0; 1; 2; 3; 4; 5+) counting the reported symptoms. Additionally, a record was made of when the blood sample was provided and whether an immunosuppression was present. For those with immunosuppression, based on a multiple-choice question, it was documented if one of the following diseases were diagnosed by a physician: metabolic disease, cancer, inflammatory rheumatic disease, chronic inflammatory respiratory disease, chronic inflammatory bowel disease, chronic nerve disease, HIV infection/AIDS as well as medication of immunosuppressants.

### 2.4. Statistics

In the descriptive analysis, continuous data were presented as mean, standard deviation (SD), minimum (Min) and maximum (Max) and categorical data as absolute numbers and percentages. For bivariate statistical analyses, non-parametric tests such as Kruskal–Wallis and Wilcoxon rank-sum tests were used. Pairwise comparisons were adjusted for multiple testing according to Bonferroni. A Tobit model [19], also known as censored regression, was used for multivariate analysis. This was because anti-spike was right/upper censored from the laboratory at 2080 BAU/mL. To meet the assumptions of the model, the anti-spike outcome variable was transformed into a logarithmic scale [20]. It was corrected for age in years, sex (reference male), immunosuppression (reference not immunosuppressed), days from the blood sample since second vaccination and vaccine combination (reference BioNTech/BioNTech). Furthermore, outliers were excluded when standardised residuals were beyond 3 and –3. These exclusions amounted to n = 6. The log estimates were exponentiated to improve comprehensibility. Variables with a contrast were labelled as relative change and relative difference for continuous variables. Exact *p*-values were displayed. Statistical analyses were performed using R version 4.1.1 (The R Foundation for Statistical Computing, Vienna, Austria).

## 3. Results

In the evaluation were included n = 545 employees. Five vaccine groupings had enough data to be examined in more detail (see Table 1). The largest group was BioNTech/BioNTech (n = 357), followed by AstraZeneca/BioNTech (n = 116), AstraZeneca/AstraZeneca (n = 37), Moderna/Moderna (n = 18) and finally AstraZeneca/Moderna (n = 17). Slightly less than one-third of the sample was male (men 27.9%; women 72.1%). The mean age was 44.91 years but was significantly lower in the AstraZeneca/Moderna (37.47) and Moderna/Moderna (39.22) groups. One-tenth (n = 10.2%) reported an existing immunosuppression in the questionnaire. Chronic inflammatory respiratory disease (n = 15), inflammatory rheumatic disease (n = 11), metabolic diseases (n = 9) and cancer (n = 7) were the most frequently reported illnesses. If the first vaccination was with an mRNA vaccine, it was more frequently reported that no side effects occurred (~23% vs. 5.9–11.1%) (Table 1). With the second vaccination, the ratio was reversed, and in the AstraZeneca/AstraZeneca group, just over half reported that no vaccination side effects occurred (Table 1).

On average, 183 days (range 130–280) lapsed between the second vaccination and the blood collection. However, the AstraZeneca/Moderna and Moderna/Moderna vaccination groupings were about 20 days below the mean. These two vaccine groupings also had the highest average anti-spike IgG values per BAU/mL in the blood, namely, Moderna/Moderna with 1454.2 and AstraZeneca/Moderna with 1114.6. BioNTech/BioNTech and AstraZeneca/BioNTech were at a similar level, with 605.8 and 530.3, respectively. AstraZeneca/AstraZeneca was significantly lower at 233.2. Overall, n = 533 (97.8%) of the anti-spike samples were above the laboratory’s reference range (<33.8 BAU/mL). In sharp contrast, n = 9 (24.3%) of the AstraZeneca/AstraZeneca group was below that limit (Table 1).

**Figure 2:**
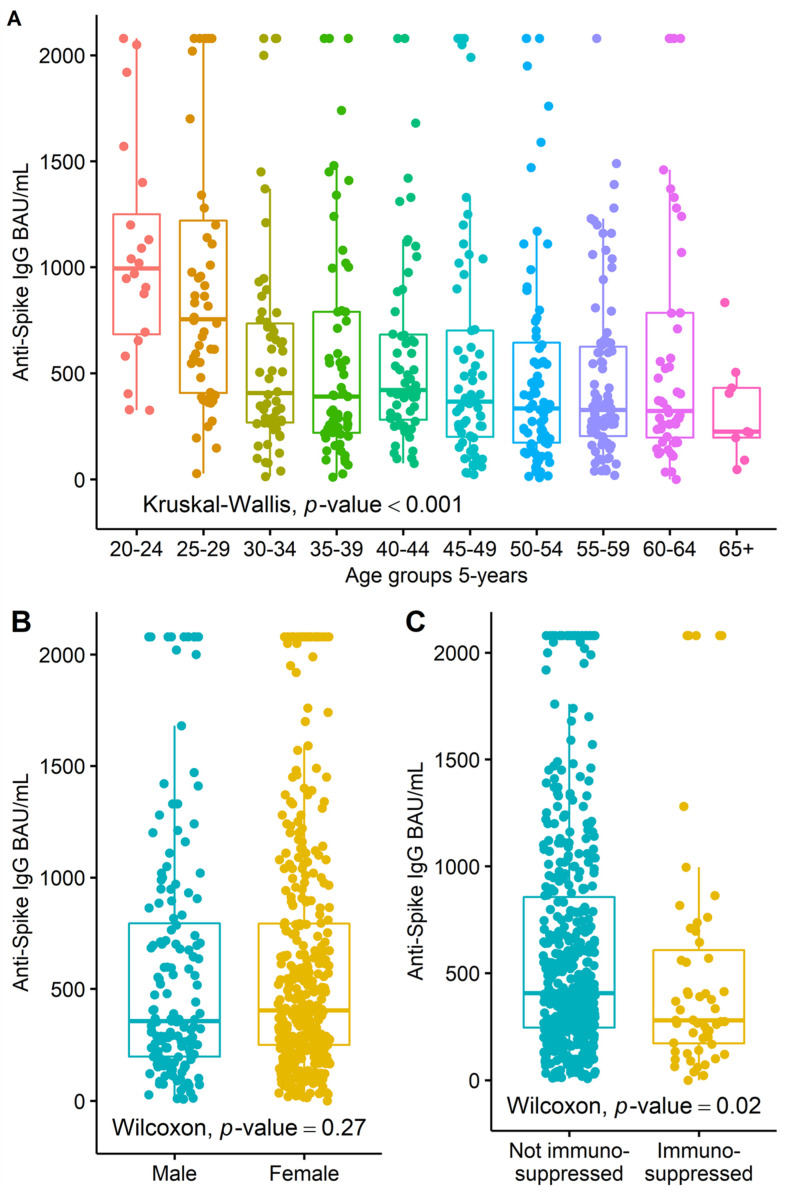
(**A**) The distribution of 5-year age groups in relation to anti-spike IgG BAU/mL. (**B**) The relation of sex to anti-spike. (**C**) The relation of immune status to anti-spike.

Figure 2A shows the level of anti-spike in relation to the 5-year age groups. The youngest age group, 20- to 24-year-olds, had the highest anti-spike value, with an average of 1059.1 BAU/mL (SD 527.0). The following age group, 25–29, had the second-highest value, with an average of 947.1 BAU/mL (SD 642.9). The value fell again in the next age group and was relatively constant at an average of 623.4 to 328.4 BAU/mL for the remaining age groups. A Kruskal–Wallis test showed a statistical difference (*p* < 0.001). Women had a slightly higher anti-spike concentration than men (see Figure 2B). Immunosuppressed hospital workers had reduced anti-spike concentrations (see Figure 2C).

**Figure 3:**
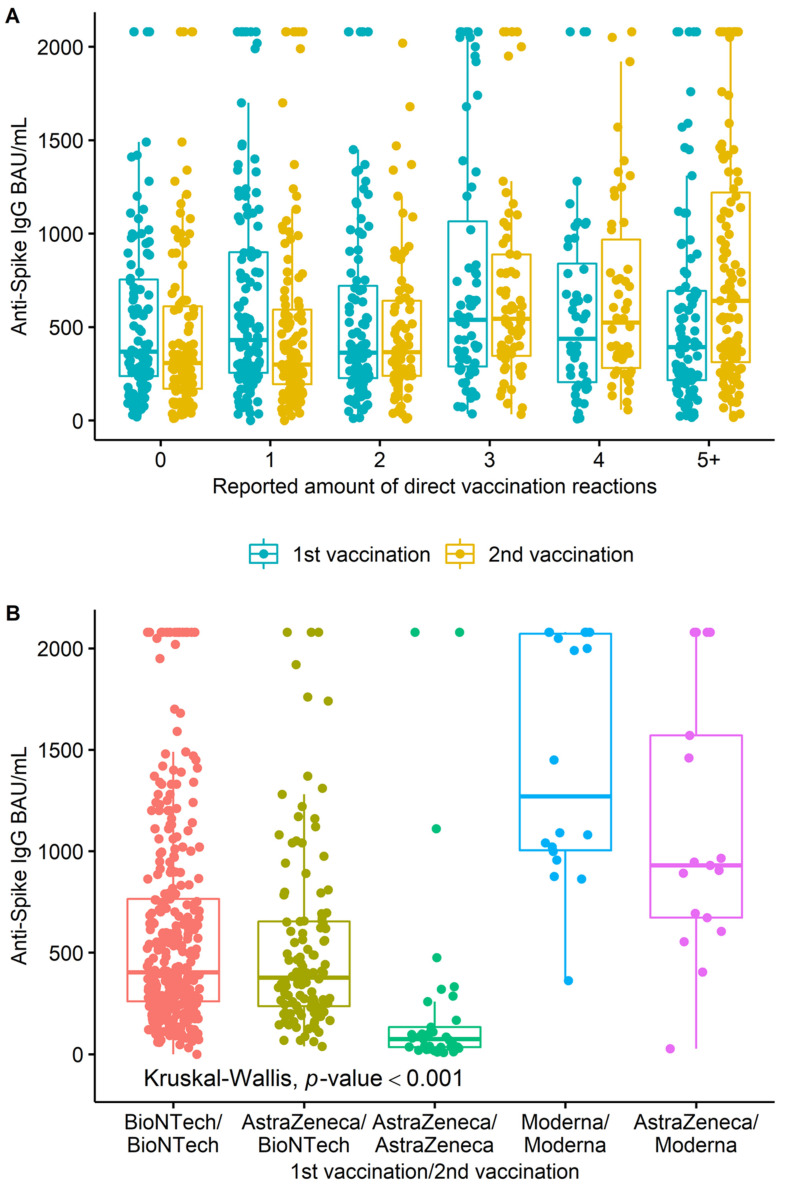
(**A**) The reported number of direct reactions after the first and second vaccinations, respectively, in relation to anti-spike IgG BAU/mL. (**B**) The reported vaccine combinations in relation to the anti-spike.

Figure 3A shows the level of the anti-spike value in relation to the reported number of side effects after the first and second vaccination. No correlation was seen for the side effects of the first vaccination (Kruskal–Wallis *p* = 0.21). However, for the side effects of the second vaccination, a correlation with the anti-spike could be seen. The more the side effects were reported, the higher were the anti-spike values (Kruskal–Wallis *p* < 0.001).

Figure 3B visualises the results of the anti-spikes from Table 1. The different levels of antibodies between the different vaccine combinations were striking. This was also confirmed by a Kruskal–Wallis test (*p* < 0.001). Subsequently, pairwise comparisons by Wilcoxon rank-sum tests after Bonferroni correction showed that each set of vaccine combinations was statistically significantly different, except AstraZeneca/Moderna and Moderna/Moderna. Table 2 displays the Tobit model with the logarithmic anti-spike as the dependent variable. Because of exclusions for statistical requirements and missing data, the sample size in this analysis amounted to n = 531. The *p*-value of the likelihood ratio test was <0.001; hence, the model differed statistically significant from a null model. Of the variance in the response variable, 35.8% could be explained by the explanatory variables. Significant impacts were present for age, immunosuppression, days between blood sample and second vaccination and for three of four vaccination schemas in contrast to BioNTech/BioNTech. The effect size of the two continuous variables accumulated with each additional day/year, respectively. With each year, about 1% less anti-spike (relative difference 0.99; 95% CI 0.98–0.99) was provable. A lack of immunosuppression (relative change 0.75; 95% CI 0.59–0.95) and fewer days between the second vaccination and the blood sample were associated with significantly higher anti-spike levels (relative difference 0.99; 95% CI 0.99–1.00). Only AstraZeneca/BioNTech did not differ in statistical significance compared to BioNTech/BioNTech (relative change 0.96; 95% CI 0.79–1.16).

**Figure 4:**
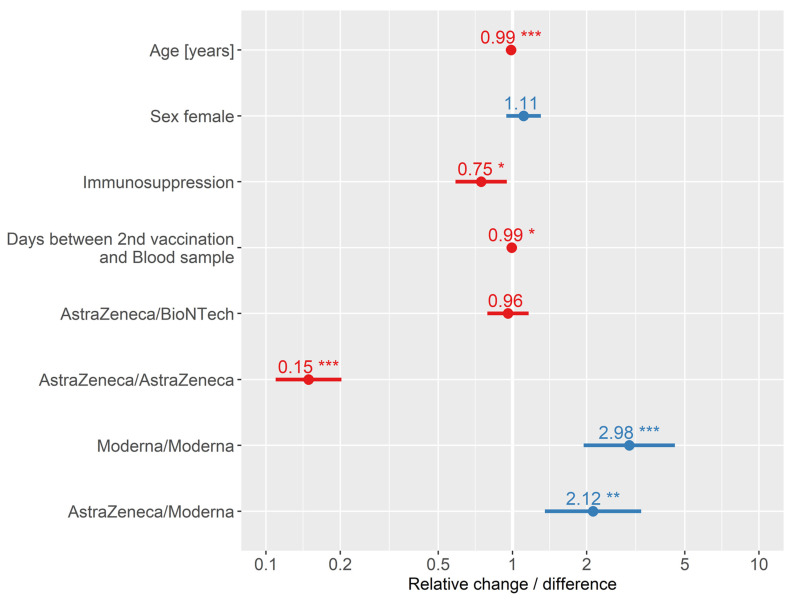
Graphic visualisation of the Tobit model/censored regression (n = 531). Blue colour represents relative change/difference to spike levels above 1 and red below. * *p* < 0.05; ** *p* < 0.01; *** *p* < 0.001.

The graphic visualisation of the Tobit model is displayed in Figure 4. The small but highly statistically significant effect of older age on lower anti-spike is visible. Immunosuppressed persons had 25% lower anti-spike values compared to those who were non-immunosuppressed. A small but significant effect of days since the second vaccination and blood sample taken could also be shown. Adjusted for all other variables in the model, recipients of AstraZeneca/AstraZeneca had only 15% of the anti-spike level of those who were vaccinated with BioNTech/BioNTech. In strict contrast, Moderna/Moderna had three times the anti-spike level and AstraZeneca/Moderna twice that of BioNTech/BioNTech.

Details are displayed in Table 2. The reference for sex is male, the reference for immunosuppression is no immunosuppression, and the reference for vaccine combination is BioNTech/BioNTech.

## 4. Discussion

In this cross-sectional study, we were able to measure the anti-spike antibody protein levels of hospital employees after the second vaccination and to study different influencing factors such as time elapsed after vaccination, vaccine schema, age, gender and immune status. After an average of 183 days following the second vaccination, the average anti-spike level was 608 BAU/mL. Additionally, as expected, immunosuppressed individuals had a lower value. The anti-spike antibody count also declined with increasing age; it was highest in the 20–24 age group and then fell steadily until a plateau appeared in middle age. There was also a correlation between the anti-spike count and the number of reported adverse events following vaccination. This was not observed with the first vaccination; however, the more side effects that were reported in the questionnaire following the second vaccination, the higher the measured anti-spike value.

The most important finding is that the anti-spike count differs depending on the vaccine schema administered. The value was lowest for AstraZeneca/AstraZeneca; nearly a quarter of individuals who received these vaccinations had levels below even the laboratory threshold. The mRNA/mRNA and AstraZeneca/mRNA vaccine combinations still had a stable anti-spike level after half a year, which was confirmed by the Tobit model.

In this study, women had a slightly higher average anti-spike level than men (men 587.13 vs. women 616.48). Although the difference was not statistically significant, other studies also confirmed this difference [21,22]. We could also show that with increased age, the anti-spike level was lower (see Table 1); this is consistent with other studies [21,23]. As expected, immunosuppressed individuals also had lower anti-spike levels (immunosuppressed 506.22 vs. non-immunosuppressed 620.63 on average). In our study and others, the data were logically consistent [21,24]. We also found that more vaccine responses were reported when the first vaccination was AstraZeneca as opposed to when it was an mRNA vaccine. This ratio reversed with the second vaccination. We also found that increased vaccine reactions following the second vaccination were associated with higher anti-spike levels. This was also reported in other studies [23,25].

The anti-spike level six months after vaccination differed significantly depending on the vaccine combination used. The vector/vector combination showed the lowest level (Ø 233.21 BAU/mL). The mRNA and vector/mRNA combinations held more than twice the anti-spike concentration. Vaccination with AstraZeneca/Moderna or Moderna/Moderna resulted in more than double and nearly triple the titres of spike antibodies compared to BioNTech/BioNTech. Steensels et al. reported higher antibody titres for Moderna as well [26]. Other studies have already shown that a significantly lower anti-spike count could be detected in the blood after a double vaccination with AstraZeneca [27,28]. However, the anti-spike for AstraZeneca/BioNTech and BioNTech/BioNTech also dropped sharply compared to what had been measured shortly after a second vaccination [27,28]. This decline happened at a faster rate than with AstraZeneca/AstraZeneca. The research objective was to discover whether sufficient vaccine protection still exists six months after a second vaccination. Since this research is ongoing, this question is not easy to answer. In addition to the spike protein antibodies that were examined, T cells play an important role. T cells seem to prevent a severe course of disease or death, whereas spike antibodies prevent infection [29,30]. One study showed that 506 BAU/mL of anti-spike was sufficient to prevent 80% of all symptomatic infections with the Alpha (B.1.1.7) variant [14]. In our sample, 57.6% (n = 314) had levels below that threshold, and 2.2% (n = 12) were even under the level set by the laboratory (<33.8 BAU/mL). A third dose of a vaccine could be advisable for everyone after half a year. In particular, people who are immunosuppressed or who received a double dose of a vector vaccine could consider being vaccinated with an mRNA vaccine.

One strength of this study is that a real-world sample of hospital staff was surveyed. The study population represents a wide spectrum of occupations and ages. Since the blood samples were analysed in a single certified laboratory and the anti-spike was measured after a relatively long period, the reliability of the measurements was ensured. Moreover, all but one participant filled out the questionnaire, and there were few missing values. The range for missing values was 0% for sex and age, and up to 6.9% for the number of directly reported reactions after vaccination. Almost half of all vaccinated hospital employees were included in the study. The staff was recruited across all sectors, though the proportion of those in the medical departments was higher. Distribution within the vaccine combinations is very heterogeneous. However, the distribution corresponds to the reality of everyday life in this hospital, also with respect to the vaccines available at the time. The study sample represented the hospital population regarding sex (27.9% vs. 26% female) and age (mean 44.9 vs. 40.6 years) well. Participation in the study was voluntary. This implies that the sample was a convenience sample. This may weaken the generalizability. Although AstraZeneca/Moderna (n = 17) and Moderna/Moderna (n = 18) had the highest anti-spike levels, these combinations were administered to few people, they were younger, and they had fewer days between the second vaccination and blood sample. However, we adjusted for these factors in the Tobit model, and they still had statistically significant differences. Blood samples had to be taken at a specific point since the administration of booster vaccinations had already started at that time. Delaying third vaccinations for the purpose of this study would not have been ethically justifiable. Though we pushed the blood collection forward, some potential subjects were lost due to their receipt of the booster vaccination. Additionally, the questionnaire only asked participants to divulge the month of the second vaccination, which was imputed to have taken place on the 15th. We examined anti-spike levels following the second vaccination in the period from summer to autumn 2021. By mid-2022, full vaccination had been achieved after two doses; this was the case for 72.2% of all people in Germany. In total, 61.6% have received their third vaccination, and a fourth vaccination is already being administered to groups at particular risk [31].

## 5. Conclusions

We were able to demonstrate differences in spike antibody levels in persons vaccinated depending on the age and vaccine schema. Additionally, the more days between the second vaccination and the blood sample, the lower the anti-spike levels. A heterogeneous vaccination with an mRNA vaccine providing anti-spike levels was found to be equivalent to two vaccinations with the same mRNA vaccine. According to the findings, it might be possible that a booster with mRNA vaccine would have better improved the anti-spike level if two vector vaccines had been administered before. This is particularly relevant for the 10.6% of people in Germany who received two vaccinations but not three, and for healthcare workers who are at a higher risk of exposure to SARS-CoV-2.

## Figures and Tables

**Table 1:** Baseline characteristics of the study population.

	AstraZeneca/ AstraZeneca (n = 37)	AstraZeneca/ BioNTech (n = 116)	AstraZeneca/ Moderna (n = 17)	BioNTech/ BioNTech (n = 357)	Moderna/ Moderna (n = 18)	Total (n = 545)
Sex						
Male	10 (27.0%)	21 (18.1%)	4 (23.5%)	111 (31.1%)	6 (33.3%)	152 (27.9%)
Female	27 (73.0%)	95 (81.9%)	13 (76.5%)	246 (68.9%)	12 (66.7%)	393 (72.1%)
Age						
Mean (SD)	49.41 (10.24)	46.48 (11.28)	37.47 (14.97)	44.57 (11.83)	39.22 (13.06)	44.91 (11.92)
Min–Max	31.00–64.00	19.00–62.00	20.00–63.00	22.00–69.00	20.00–59.00	19.00–69.00
Immunosuppression						
Not immunosuppressed	36 (97.3%)	100 (89.3%)	16 (94.1%)	314 (89.0%)	16 (88.9%)	482 (89.8%)
Immunosuppressed	1 (2.7%)	12 (10.7%)	1 (5.9%)	39 (11.0%)	2 (11.1%)	55 (10.2%)
n-missing	0	4	0	4	0	8
1st vaccination: number of directly reported reactions						
0	4 (11.1%)	12 (10.4%)	1 (5.9%)	82 (23.1%)	4 (22.2%)	103 (19.0%)
1	7 (19.4%)	10 (8.7%)	2 (11.8%)	97 (27.3%)	8 (44.4%)	124 (22.9%)
2	6 (16.7%)	20 (17.4%)	1 (5.9%)	74 (20.8%)	3 (16.7%)	104 (19.2%)
3	4 (11.1%)	13 (11.3%)	2 (11.8%)	47 (13.2%)	2 (11.1%)	68 (12.6%)
4	5 (13.9%)	13 (11.3%)	2 (11.8%)	28 (7.9%)	0 (0.0%)	48 (8.9%)
5+	10 (27.8%)	47 (40.9%)	9 (52.9%)	27 (7.6%)	1 (5.6%)	94 (17.4%)
n-missing	1	1	0	2	0	4
2nd vaccination: number of directly reported reactions						
0	18 (48.6%)	21 (18.1%)	1 (5.9%)	65 (18.2%)	2 (11.1%)	107 (19.6%)
1	7 (18.9%)	28 (24.1%)	2 (11.8%)	79 (22.1%)	3 (16.7%)	119 (21.8%)
2	7 (18.9%)	14 (12.1%)	4 (23.5%)	51 (14.3%)	3 (16.7%)	79 (14.5%)
3	2 (5.4%)	16 (13.8%)	2 (11.8%)	44 (12.3%)	3 (16.7%)	67 (12.3%)
4	1 (2.7%)	15 (12.9%)	1 (5.9%)	32 (9.0%)	1 (5.6%)	50 (9.2%)
5+	2 (5.4%)	22 (19.0%)	7 (41.2%)	86 (24.1%)	6 (33.3%)	123 (22.6%)
Days between the second vaccination and blood sample						
MW (SD)	191.27 (4.91)	190.78 (6.24)	164.18 (8.14)	180.94 (13.29)	166.67 (10.31)	182.74 (13.07)
Min–Max	163.00–195.00	161.00–199.00	160.00–195.00	130.00–280.00	160.00–193.00	130.00–280.00
Coronavirus SARS-CoV-2 spike IgG BAU/ml						
MW (SD)	233.21 (488.91)	530.28 (453.27)	1114.56 (655.04)	605.76 (516.13)	1454.22 (587.28)	608.29 (547.30)
Min–Max	8.10–2080.00	38.80–2080.00	27.60–2080.00	0.00–2080.00	362.00–2080.00	0.00–2080.00
Reference range spike IgG						
<33.8	9 (24.3%)	0 (0.0%)	1 (5.9%)	2 (0.6%)	0 (0.0%)	12 (2.2%)
≥33.8	28 (75.7%)	116 (100.0%)	16 (94.1%)	355 (99.4%)	18 (100.0%)	533 (97.8%)

**Table 2:** Tobit model with logarithmic anti-spike IgG BAU/mL as the outcome variable. The relative change/difference, lower limit (LL) and upper limit (UL) were exponentiated from the estimate log (n = 531).

Coefficients	Reference	Estimate Log	Std. Error	z Value	Relative Change/ Difference	95% CI		*p*-Value
						LL	UL	
Age	-	–0.014	0.003	–4.462	0.99	0.98	0.99	<0.001
Sex	Male	0.104	0.082	1.267	1.11	0.94	1.30	0.205
Immunosuppressed	Not immunosuppressed	–0.292	0.122	–2.391	0.75	0.59	0.95	0.017
Days between blood samples and 2nd vaccination	-	–0.007	0.003	–1.971	0.99	0.99	1.00	0.049
1st/2nd vaccination								
- AstraZeneca/BioNTech	BioNTech/BioNTech	–0.042	0.099	–0.422	0.96	0.79	1.16	0.673
- AstraZeneca/AstraZeneca		–1.904	0.157	–12.159	0.15	0.11	0.20	<0.001
- Moderna/Moderna		1.091	0.217	5.020	2.98	1.94	4.56	0.001
- AstraZeneca/Moderna		0.753	0.230	3.279	2.12	1.35	3.33	<0.001
Intercept		7.910	0.648	12.216	-	-	-	<0.001

LR chi^2^ (8) 227.49 Prob > chi^2^ < 0.001 Pseudo R^2^ 0.358, sigma 0.84.

## Data Availability

The data presented in this study are available upon request from the corresponding author.

## Supplementary Materials

The following supporting information can be downloaded at: https://www.mdpi.com/article/10.3390/ijerph191811422/s1, S1: laboratory instructions; S2: questionnaire. References [32,33,34,35,36,37,38,39,40,41,42,43,44,45,46,47,48,49] are cited in the supplementary materials.
