# Supplementary material for: Coronavirus Vaccination: Spike Antibody Levels in Health Workers after Six Months—A Cross-Sectional Study
Source: Int J Environ Res Public Health. 2022 Sep 10;19(18):11422. doi: 10.3390/ijerph191811422 (PMC9517660; doi:10.3390/ijerph191811422)
Supplement: Supplementary file 1 [file ijerph-19-11422-s001.zip › ijerph-1878113-supplementary/S1 laboratory instructions.pdf]

en

CoV-2 IgG
6R86
H07891R03
B6R860

SARS-CoV-2 IgG

FOR USE WITH
ARCHITECT

Revised May 2020.

REF 6R86-22
REF 6R86-32

Instructions must be carefully followed. Reliability of assay results cannot be guaranteed if there are any deviations from these instructions.

For laboratory professional use only.

NAME

SARS-CoV-2 IgG (also referred to as CoV-2 IgG)

INTENDED USE

The SARS-CoV-2 IgG assay is a chemiluminescent microparticle immunoassay (CMIA) used for the qualitative detection of IgG antibodies to SARS-CoV-2 in human serum and plasma on the ARCHITECT i System.

The SARS-CoV-2 IgG assay is to be used as an aid in the diagnosis of SARS-CoV-2 infection in conjunction with clinical presentation and other laboratory tests. Results from the SARS-CoV-2 IgG assay should not be used as the sole basis for diagnosis.

SUMMARY AND EXPLANATION OF THE TEST

The SARS-CoV-2 IgG assay is designed to detect immunoglobulin class G (IgG) antibodies to the nucleocapsid protein of SARS-CoV-2 in serum and plasma from patients with signs and symptoms of infection who are suspected of coronavirus disease (COVID-19) or in serum and plasma of subjects that may have been infected by SARS-CoV-2.

COVID-19 is defined as illness caused by a novel coronavirus now called severe acute respiratory syndrome coronavirus 2 (SARS-CoV-2, formerly called 2019-nCoV), which was first identified in December 2019 during an outbreak of respiratory illness cases in China.[1] On March 11, 2020, the World Health Organization (WHO) declared COVID-19 a global pandemic.[2] The incubation period of COVID-19 ranges between 1 and 14 days, with the majority of cases manifesting within 3 to 5 days. The most common symptoms of COVID-19 are fever, tiredness, dry cough, and difficulty breathing. A severe acute respiratory distress syndrome (ARDS) may develop.[3] The case fatality rate is reported at 6.3% globally[4] and increases with age and comorbidities.

The causative agent of COVID-19 is a beta coronavirus and belongs to a family of viruses that may cause respiratory symptoms ranging from common cold to severe pneumonia. These viruses are common in animals worldwide and may eventually spill over to humans, as has likely happened with SARS-CoV-2.[1]

The host immune system reacts to the infection by SARS-CoV-2 by producing specific antibodies. These antibodies have been reported to appear in serum or plasma of infected individuals after the detection of viral ribonucleic acid (RNA) in swabs[5] and a few days to 2 weeks after the onset of symptoms.[6] Specific IgG antibodies to SARS-CoV-2 are detectable in COVID-19 patients during the symptomatic phase of the disease after RNA is no longer detectable.[5, 6] The sensitivity of combining RNA with antibody results has been reported as > 99%.[5] The persistence of IgG antibodies allows identification of people who have been infected in the past, recovered from the illness, and possibly become immune.[7] IgG detection and other serological assays will play an important role in research and surveillance.[8]

BIOLOGICAL PRINCIPLES OF THE PROCEDURE

This assay is an automated, two-step immunoassay for the qualitative detection of IgG antibodies to SARS-CoV-2 in human serum and plasma using chemiluminescent microparticle immunoassay (CMIA) technology.

Sample, SARS-CoV-2 antigen coated paramagnetic microparticles, and assay diluent are combined and incubated. The IgG antibodies to SARS-CoV-2 present in the sample bind to the SARS-CoV-2 antigen coated microparticles. The mixture is washed. Anti-human IgG acridinium-labeled conjugate is added to create a reaction mixture and incubated. Following a wash cycle, Pre-Trigger and Trigger Solutions are added.

The resulting chemiluminescent reaction is measured as a relative light unit (RLU). There is a direct relationship between the amount of IgG antibodies to SARS-CoV-2 in the sample and the RLU detected by the system optics.

This relationship is reflected in the calculated Index (S/C).

The presence or absence of IgG antibodies to SARS-CoV-2 in the sample is determined by comparing the chemiluminescent RLU in the reaction to the calibrator RLU.

For additional information on system and assay technology, refer to the ARCHITECT System Operations Manual, Section 3.

REAGENTS

Kit Contents

SARS-CoV-2 IgG Reagent Kit 6R86

NOTE: Some kit sizes may not be available for use on all ARCHITECT i Systems. Please contact your local distributor.

Volumes (mL) listed in the following table indicate the volume per bottle.

REF	6R86-22	6R86-32
Tests per kit	100	500
Number of kits per box	1	1
Tests per box	100	500
MICROPARTICLES	6.6 mL	27.0 mL
CONJUGATE	5.8 mL	26.2 mL
ASSAY DILUENT	7.9 mL	40.7 mL

MICROPARTICLES Purified SARS-CoV-2 recombinant antigen coated microparticles in TRIS buffer with surfactant. Minimum concentration: 0.045% solids. Preservatives: ProClin 950 and sodium azide.

CONJUGATE Anti-human IgG (mouse, monoclonal) acridinium-labeled conjugate in MES buffer with surfactant and protein (bovine) stabilizer. Minimum concentration: 4 ng/mL. Preservatives: ProClin 300 and antimicrobial agents.

ASSAY DILUENT TRIS buffer and detergent. Preservatives: ProClin 950 and sodium azide.

Warnings and Precautions

- IVD
- For *In Vitro* Diagnostic Use

Abbott

Safety Precautions

CAUTION: This product requires the handling of human specimens. It is recommended that all human-sourced materials and all consumables contaminated with potentially infectious materials be considered potentially infectious and handled in accordance with the OSHA Standard on Bloodborne Pathogens. Biosafety Level 2 or other appropriate regional, national, and institutional biosafety practices should be used for materials that contain, are suspected of containing, or are contaminated with infectious agents.[9-12]

The following warnings and precautions apply to: MICROPARTICLES and ASSAY DILUENT	
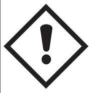	
**WARNING**	Contains methylisothiazolone and sodium azide.
H317	May cause an allergic skin reaction.
EUH032	Contact with acids liberates very toxic gas.
**Prevention**	
P261	Avoid breathing mist / vapors / spray.
P272	Contaminated work clothing should not be allowed out of the workplace.
P280	Wear protective gloves / protective clothing / eye protection.
**Response**	
P302+P352	IF ON SKIN: Wash with plenty of water.
P333+P313	If skin irritation or rash occurs: Get medical advice / attention.
P362+P364	Take off contaminated clothing and wash it before reuse.
**Disposal**	
P501	Dispose of contents / container in accordance with local regulations.

The following warnings and precautions apply to: CONJUGATE	
H402*	Harmful to aquatic life.
H412	Harmful to aquatic life with long lasting effects.
**Prevention**	
P273	Avoid release to the environment.
**Disposal**	
P501	Dispose of contents / container in accordance with local regulations.

* Not applicable where regulation EC 1272/2008 (CLP) has been implemented.

Follow local chemical disposal regulations based on your location along with recommendations and content in the Safety Data Sheet to determine the safe disposal of this product.

For the most current hazard information, see the product Safety Data Sheet.

Safety Data Sheets are available at www.corelaboratory.abbott or contact your local representative.

For a detailed discussion of safety precautions during system operation, refer to the ARCHITECT System Operations Manual, Section 8.

Reagent Handling

- Reagents are shipped on wet ice.
- Do not pool reagents within a kit or between kits.
- Before loading the reagent kit on the system for the first time, the microparticle bottle requires mixing to resuspend microparticles that may have settled during shipment. For microparticle mixing instructions, refer to the PROCEDURE, Assay Procedure section of this package insert.
- **Septums MUST be used to prevent reagent evaporation and contamination and to ensure reagent integrity. Reliability of assay results cannot be guaranteed if septums are not used according to the instructions in this package insert.**
  - To avoid contamination, wear clean gloves when placing a septum on an uncapped reagent bottle.
  - Once a septum has been placed on an open reagent bottle, do not invert the bottle as this will result in reagent leakage and may compromise assay results.
  - Over time, residual liquids may dry on the septum surface. These are typically dried salts and have no effect on assay efficacy.
- When handling conjugate vials, change gloves that have contacted human serum or plasma, since introduction of human IgG will result in a neutralized conjugate.

For a detailed discussion of reagent handling precautions during system operation, refer to the ARCHITECT System Operations Manual, Section 7.

Reagent Storage

- Do not freeze.
- Protect from light.

	Storage Temperature	Maximum Storage Time	Additional Storage Instructions
**Unopened**	2 to 8°C	Until expiration date	Store in upright position.
**Onboard**	System Temperature	7 days	
**Opened**	2 to 8°C	Until expiration date	Store in upright position. If the microparticle bottle does not remain upright (with a septum installed) while in refrigerated storage off the system, the reagent kit must be discarded.

Reagents may be stored on or off the ARCHITECT i System. If reagents are removed from the system, store them at 2 to 8°C (with septums and replacement caps) in an upright position. For reagents stored off the system, it is recommended that they be stored in their original trays and boxes to ensure they remain upright.

For information on unloading reagents, refer to the ARCHITECT System Operations Manual, Section 5.

Indications of Reagent Deterioration

Deterioration of the reagents may be indicated when a calibration error occurs or a control value is out of the specified range. Associated test results are invalid, and samples must be retested. Assay recalibration may be necessary.

For troubleshooting information, refer to the ARCHITECT System Operations Manual, Section 10.

INSTRUMENT PROCEDURE

The SARS-CoV-2 IgG assay file must be installed on the ARCHITECT i System prior to performing the assay.

For detailed information on assay file installation and viewing and editing assay parameters, refer to the ARCHITECT System Operations Manual, Section 2.

For information on printing assay parameters, refer to the ARCHITECT System Operations Manual, Section 5.

For a detailed description of system procedures, refer to the ARCHITECT System Operations Manual.

SPECIMEN COLLECTION AND PREPARATION FOR ANALYSIS

Specimen Types

The specimen types listed below may be used with this assay.

Specimen Types	Collection Tubes
Serum	Serum
Plasma	EDTA

- Performance has not been established for the use of cadaveric specimens or the use of bodily fluids other than human serum/plasma.
- Liquid anticoagulants may have a dilution effect resulting in lower Index (S/C) values for individual specimens.

The instrument does not provide the capability to verify specimen types. It is the responsibility of the operator to verify that the correct specimen types are used in the assay.

Specimen Conditions

- Do not use:
  - heat-inactivated specimens
  - pooled specimens
  - grossly hemolyzed specimens
  - specimens with obvious microbial contamination
  - specimens with fungal growth
- For accurate results, serum and plasma specimens should be free of fibrin, red blood cells, and other particulate matter. Serum specimens from patients receiving anticoagulant or thrombolytic therapy may contain fibrin due to incomplete clot formation.
- To prevent cross contamination, use of disposable pipettes or pipette tips is recommended.

Preparation for Analysis

- Follow the tube manufacturer's processing instructions for collection tubes. Gravity separation is not sufficient for specimen preparation.
- Specimens should be free of bubbles. Remove bubbles with an applicator stick before analysis. Use a new applicator stick for each specimen to prevent cross contamination.

To ensure consistency in results, recentrifuge specimens prior to testing if

- they contain fibrin, red blood cells, or other particulate matter.

NOTE: If fibrin, red blood cells, or other particulate matter are observed, mix by low speed vortex or by inverting 10 times prior to recentrifugation.

Prepare frozen specimens as follows:

- Frozen specimens must be completely thawed before mixing.
- Mix thawed specimens thoroughly by low speed vortex or by inverting 10 times.
- Visually inspect the specimens. If layering or stratification is observed, mix until specimens are visibly homogeneous.
- If specimens are not mixed thoroughly, inconsistent results may be obtained.
- Recentrifuge specimens.

Recentrifugation of Specimens

- Transfer specimens to a centrifuge tube and centrifuge.
- Transfer clarified specimen to a sample cup or secondary tube for testing. For centrifuged specimens with a lipid layer, transfer only the clarified specimen and not the lipemic material.

Specimen Storage

Specimen Type	Temperature	Maximum Storage Time
Serum/Plasma	Room temperature (15 to 30°C)	2 days
	2 to 8°C	7 days

If testing will be delayed more than 7 days, it is recommended to store frozen.

It is the responsibility of the individual laboratory to determine specific specimen stability criteria for their laboratory per their laboratory workflow.

For additional information on sample handling and processing, refer to CLSI GP44-A4.[13] The storage information provided here is based on data maintained by the manufacturer.

Frozen specimens subjected to up to 2 freeze/thaw cycles have been evaluated.

Specimen Shipping

Package and label specimens in compliance with applicable state, federal, and international regulations covering the transport of clinical specimens and infectious substances.

Do not exceed the storage limitations listed above.

PROCEDURE

Materials Provided

6R86 SARS-CoV-2 IgG Reagent Kit

Materials Required but not Provided

- SARS-CoV-2 IgG assay file found on www.corelaboratory.abbott
- 6R86-02 SARS-CoV-2 IgG Calibrator Kit
- 6R86-12 SARS-CoV-2 IgG Control Kit or other control material containing IgG antibodies to SARS-CoV-2
- ARCHITECT Pre-Trigger Solution
- ARCHITECT Trigger Solution
- ARCHITECT Wash Buffer
- ARCHITECT Septum

For information on materials required for operation of the instrument, refer to the ARCHITECT System Operations Manual, Section 1.

For information on materials required for maintenance procedures, refer to the ARCHITECT System Operations Manual, Section 9.

Assay Procedure

For a detailed description of how to run an assay, refer to the ARCHITECT System Operations Manual, Section 5.

- If using primary or aliquot tubes, refer to the ARCHITECT System Operations Manual, Section 5 to ensure sufficient specimen is present.
- Minimum sample cup volume is calculated by the system and printed on the Order List report. To minimize the effects of evaporation, verify adequate sample cup volume is present prior to running the test.
- Before loading the reagent kit on the system for the first time, the microparticle bottle requires mixing to resuspend microparticles that may have settled during shipment. After the first time the microparticles have been loaded, no further mixing is required.
  - **Invert the microparticle bottle 30 times.**
  - Visually inspect the bottle to ensure microparticles are resuspended. If microparticles are still adhered to the bottle, continue to invert the bottle until the microparticles have been completely resuspended.
  - **If the microparticles do not resuspend, DO NOT USE. Contact your local Abbott representative.**
  - Once the microparticles have been resuspended, place a septum on the bottle. For instructions about placing septums on bottles, refer to the Reagent Handling section of this package insert.

- Maximum number of replicates sampled from the same sample cup: 10
  - Priority:
    - Sample volume for first test: 75 µL
    - Sample volume for each additional test from same sample cup: 25 µL
  - Routine:
    - Sample volume for first test: 150 µL
    - Sample volume for each additional test from same sample cup: 25 µL
- Refer to the SARS-CoV-2 IgG calibrator package insert REF 6R86-02 and/or SARS-CoV-2 IgG control package insert REF 6R86-12 for preparation and usage.
- For general operating procedures, refer to the ARCHITECT System Operations Manual, Section 5.
- For optimal performance, it is important to perform routine maintenance as described in the ARCHITECT System Operations Manual, Section 9. Perform maintenance more frequently when required by laboratory procedures.

Sample Dilution Procedures

Dilution of samples for the SARS-CoV-2 IgG assay has not been verified.

Calibration

For instructions on performing a calibration, refer to the ARCHITECT System Operations Manual, Section 6.

Each assay control must be tested to evaluate the assay calibration.

Once a calibration is accepted and stored, all subsequent samples may be tested without further calibration unless:

- A reagent kit with a new lot number is used.
- Daily quality control results are outside of quality control limits used to monitor and control system performance.

This assay may require recalibration after maintenance to critical parts or subsystems or after service procedures have been performed.

Quality Control Procedures

The recommended control requirement for the SARS-CoV-2 IgG assay is that a single sample of each control level be tested once every 24 hours each day of use.

Additional controls may be tested in accordance with local, state, and/or federal regulations or accreditation requirements and your laboratory's quality control policy.

To establish statistically-based control limits, each laboratory should establish its own concentration target and ranges for new control lots at each clinically relevant control level. This can be accomplished by assaying a minimum of 20 replicates over several (3-5) days and using the reported results to establish the expected average (target) and variability about this average (range) for the laboratory. Sources of variation that should be included in this study in order to be representative of future system performance include:

- Multiple stored calibrations
- Multiple reagent lots
- Multiple calibrator lots
- Multiple processing modules (if applicable)
- Data points collected at different times of the day

Refer to published guidelines for information or general control recommendation, for example Clinical and Laboratory Standards Institute (CLSI) Guideline C24, 4th ed., or other published guidelines, for general quality control recommendations.[14]

- If more frequent control monitoring is required, follow the established quality control procedures for your laboratory.
- If quality control results do not meet the acceptance criteria defined by your laboratory, sample results may be suspect. Follow the established quality control procedures for your laboratory. Recalibration may be necessary. For troubleshooting information, refer to the ARCHITECT System Operations Manual, Section 10.
- Review quality control results and acceptance criteria following a change of reagent or calibrator lot.

Controls should be used according to the guidelines and recommendations of the control manufacturer. Concentration ranges provided in the control package insert should be used only for guidance.

For any control material in use, the laboratory should ensure that the matrix of the control material is suitable for use in the assay per the assay package insert.

Quality Control Guidance

Refer to "Basic QC Practices" by James O Westgard, Ph.D. for guidance on laboratory quality control practices.[15]

Verification of Assay Claims

For protocols to verify package insert claims, refer to the ARCHITECT System Operations Manual, Appendix B.

The SARS-CoV-2 IgG assay belongs to method group 5. Functional sensitivity does not apply.

RESULTS

Calculation

The ARCHITECT i System calculates the calibrator mean chemiluminescent signal from 3 calibrator replicates and stores the result. Results are reported by dividing the sample result by the stored calibrator result. The default result unit for the SARS-CoV-2 IgG assay is Index (S/C).

Interpretation of Results

The cutoff is 1.4 Index (S/C).

As with all analyte determinations, the result should be used in conjunction with information available from clinical evaluation and other diagnostic procedures.

Index (S/C)	Interpretation
< 1.4	Negative
≥ 1.4	Positive

Flags

Some results may contain information in the Flags field. For a description of the flags that may appear in this field, refer to the ARCHITECT System Operations Manual, Section 5.

LIMITATIONS OF THE PROCEDURE

- Results should be used in conjunction with other data; e.g., symptoms, results of other tests, and clinical impressions.
- Negative results do not rule out SARS-CoV-2 infection, particularly in those who have been in contact with the virus. Follow-up testing with a molecular diagnostic should be considered to rule out infection in these individuals.
- Results from antibody testing should not be used as the sole basis to diagnose or exclude SARS-CoV-2 infection or to inform infection status.
- Non-SARS-CoV-2 coronavirus strains, such as coronavirus HKU1, NL63, OC43, or 229E, have not been evaluated with this assay. In a population of patients with non-COVID-19 respiratory illnesses, no cross-reactivity has been observed. Refer to the SPECIFIC PERFORMANCE CHARACTERISTICS section of this package insert.

- Not to be used to screen units of blood for SARS-CoV-2 infection.
- Immunocompromised patients who have COVID-19 may have a delayed antibody response and produce levels of antibody which may not be detected as positive by the assay.
- Potentially interfering disease states and other cross reactants have been evaluated and are represented in the SPECIFIC PERFORMANCE CHARACTERISTICS section of this package insert.
- Specimens from patients who have received preparations of mouse monoclonal antibodies for diagnosis or therapy may contain human anti-mouse antibodies (HAMA). Such specimens may show either falsely elevated or depressed values when tested with assay kits such as SARS-CoV-2 IgG that employ mouse monoclonal antibodies.[16, 17]
- Heterophilic antibodies in human serum can react with reagent immunoglobulins, interfering with *in vitro* immunoassays. Patients routinely exposed to animals or to animal serum products can be prone to this interference, and anomalous values may be observed.[18]
- Rheumatoid factor (RF) in human serum can react with reagent immunoglobulins, interfering with *in vitro* immunoassays.[18]

SPECIFIC PERFORMANCE CHARACTERISTICS

Representative performance data are provided in this section. Results obtained in individual laboratories may vary.

Precision

Within-Laboratory Precision

Testing was conducted using 1 lot of the SARS-CoV-2 IgG Reagent Kit, 1 lot of the SARS-CoV-2 IgG Calibrator Kit, and 1 lot of the SARS-CoV-2 IgG Control Kit and 1 instrument. Two controls were assayed in replicates of 10 on 5 different days.

		Mean	Within-Run (Repeatability)		Within-Laboratory[a]	
Sample	n	(Index [S/C])	SD	%CV	SD	%CV
Negative Control	50	0.04	0.002	5.9	0.002	5.9
Positive Control	50	3.53	0.040	1.1	0.042	1.2

[a] Includes within-run and between-day variability.

Analytical Specificity

The SARS-CoV-2 IgG assay was evaluated for potential cross-reactivity from individuals with other medical conditions. A total of 182 specimens from 36 different categories were tested. One hundred eighty-one (181) specimens were negative and 1 specimen was positive by the SARS-CoV-2 IgG assay. The data are summarized in the following table. Bold indicates other respiratory illness categories.

Category	n	Positive	Negative
**Adenovirus**	**5**	**0**	**5**
Antinuclear Antibody (ANA)	5	0	5
Autoimmune Hepatitis	5	0	5
Cytomegalovirus (CMV) IgG	5	1	4
CMV Immunoglobulin Class M (IgM)	5	0	5
Double-Stranded Deoxyribonucleic Acid (dsDNA) Antibody	5	0	5
Epstein-Barr Virus (EBV) IgG	5	0	5
EBV IgM	5	0	5
Escherichia coli (E. coli) Antibody	5	0	5
HAMA	5	0	5
Hemodialysis Patients	5	0	5
Hepatitis A Virus (HAV)	5	0	5
Hepatitis B Core (HBc) IgM	4	0	4
Hepatitis B Virus (HBV)	5	0	5
Hepatitis C Virus (HCV)	5	0	5
Hepatitis D Virus (HDV)	5	0	5
Herpes Simplex Virus (HSV)	5	0	5
Heterophilic Antibody Positive	5	0	5
Human Immunodeficiency Virus (HIV)	5	0	5
Human T-Lymphotropic Virus (HTLV) Type 1	5	0	5
HTLV Type 2	5	0	5
**Influenza A**	**7**	**0**	**7**
**Influenza B**	**5**	**0**	**5**
**Influenza (Type Unknown)**	**8**	**0**	**8**
Influenza Vaccine	5	0	5
Lupus	5	0	5
Monoclonal Hyper IgG	5	0	5
**Picornavirus**	**5**	**0**	**5**
Polyclonal Hyper IgG	3	0	3
Pregnant Females	5	0	5
Pregnant Females, Multiparous	5	0	5
**Respiratory Syncytial Virus (RSV)**	**5**	**0**	**5**
RF	5	0	5
Rubella IgG	5	0	5
Toxoplasmosis IgG	5	0	5
Varicella Zoster Virus	5	0	5
Total	182	1	181

Clinical Performance

A study was performed to determine the clinical performance of the SARS-CoV-2 IgG assay.

To estimate the positive percent agreement (PPA), 122 serum and plasma specimens were collected at different times from 31 subjects who tested positive for SARS-CoV-2 by a polymerase chain reaction (PCR) method and who also presented with COVID-19 symptoms. Each specimen was tested using the SARS-CoV-2 IgG assay. The PPA and the 95% confidence interval (CI) were calculated.

To estimate the negative percent agreement (NPA), 1070 serum and plasma specimens from subjects assumed to be negative for SARS-CoV-2 were tested. Of the 1070 specimens, 997 specimens were collected prior to September 2019 (pre-COVID-19 outbreak). An additional 73 specimens were collected in 2020 from subjects who were exhibiting signs of respiratory illness but tested negative for SARS-CoV-2 by a PCR method. All 1070 specimens were tested using the SARS-CoV-2 IgG assay. The NPA and the 95% CI were calculated.

The results of both groups are presented in the following 2 tables.

Positive Agreement by Days Post-Symptom Onset

Days Post-Symptom Onset	n	Positive	Negative	PPA (95% CI)
< 3	4	0	4	0.00% (0.00, 60.24)
3 - 7	8	2	6	25.00% (3.19, 65.09)
8 - 13	22	19	3	86.36% (65.09, 97.09)
**≥ 14**	**88[a]**	**88**	**0**	**100.00% (95.89, 100.00)**

[a] Five specimens from 1 immunocompromised patient were excluded from the study. Refer to the LIMITATIONS OF THE PROCEDURE section of this package insert for further information. When the results from these specimens were included, the PPA at ≥ 14 days post-symptom onset was 96.77% (95% CI: 90.86, 99.33).

Negative Agreement by Category

Category	n	Positive	Negative	NPA (95% CI)
Pre-COVID-19 Outbreak	997	4	993	99.60% (98.98, 99.89)
Other Respiratory Illness	73	0	73	100.00% (95.07, 100.00)
**Total**	**1070**	**4**	**1066**	**99.63% (99.05, 99.90)**

Abbott

Class Specificity

The anti-human IgG antibody used in the SARS-CoV-2 IgG assay demonstrates class-specific reactivity only to human IgG isotypes. No binding interactions were observed to human IgM, human IgA, or sheep (ovine) IgG.

BIBLIOGRAPHY

1. Zhu N, Zhang D, Wang W, et al. A novel coronavirus from patients with pneumonia in China, 2019. *N Engl J Med* 2020;382:727-733. doi:10.1056/NEJMoa2001017
2. World Health Organization. *WHO Director-General's opening remarks at the media briefing on COVID-19 - 11 March 2020.* Accessed April 14, 2020. https://www.who.int/dg/speeches/detail/who-director-general-s-opening-remarks-at-the-media-briefing-on-covid-19---11-march-2020
3. World Health Organization. *Report of the WHO-China Joint Mission on Coronavirus Disease 2019 (COVID-19) 16-24 February 2020.* Accessed April 14, 2020. https://www.who.int/docs/default-source/coronaviruse/who-china-joint-mission-on-covid-19-final-report
4. World Health Organization. *Coronavirus disease 2019 (COVID-19) situation report-85, Data as received by WHO from national authorities by 10:00 CET, 14 April 2020.* Accessed April 14, 2020. https://www.who.int/docs/default-source/coronaviruse/situation-reports/20200414-sitrep-85-covid-19
5. Zhao J, Yuan Q, Wang H, et al. Antibody responses to SARS-CoV-2 in patients of novel coronavirus disease 2019. *Clin Inf Dis* Accepted manuscript. Published online 28 March 2020. doi:10.1093/cid/ciaa344
6. Xiao AT, Gao C, Zhang S. Profile of specific antibodies to SARS-CoV-2: the first report. *J Infect* 2020. doi:10.1016/j.jinf.2020.03.012
7. Shen C, Wang Z, Zhao F, et al. Treatment of 5 critically ill patients with COVID-19 with convalescent plasma. *JAMA* Published online March 27, 2020. doi:10.1001/jama.2020.4783
8. World Health Organization. *Laboratory testing strategy recommendations for COVID-19: interim guidance, 21 March 2020.* World Health Organization; 2020.
9. US Department of Labor, Occupational Safety and Health Administration, 29 CFR Part 1910.1030, Bloodborne pathogens.
10. US Department of Health and Human Services. *Biosafety in Microbiological and Biomedical Laboratories*. 5th ed. Washington, DC: US Government Printing Office; December 2009.
11. World Health Organization. *Laboratory Biosafety Manual*. 3rd ed. Geneva: World Health Organization; 2004.
12. Clinical and Laboratory Standards Institute (CLSI). *Protection of Laboratory Workers From Occupationally Acquired Infections; Approved Guideline—Fourth Edition*. CLSI Document M29-A4. Wayne, PA: CLSI; 2014.
13. Clinical and Laboratory Standards Institute (CLSI). *Procedures for the Handling and Processing of Blood Specimens for Common Laboratory Tests; Approved Guideline—Fourth Edition.* CLSI Document GP44-A4. Wayne, PA: CLSI; 2010.
14. Clinical and Laboratory Standards Institute (CLSI). *Statistical Quality Control for Quantitative Measurement Procedures: Principles and Definitions.* 4th ed. CLSI Guideline C24. Wayne, PA: CLSI; 2016.
15. Westgard JO. *Basic QC Practices*. 3rd ed. Madison, WI: Westgard Quality Corporation; 2010.
16. Primus FJ, Kelley EA, Hansen HJ, et al. "Sandwich"-type immunoassay of carcinoembryonic antigen in patients receiving murine monoclonal antibodies for diagnosis and therapy. *Clin Chem* 1988;34(2):261-264.
17. Schroff RW, Foon KA, Beatty SM, et al. Human anti-murine immunoglobulin responses in patients receiving monoclonal antibody therapy. *Cancer Res* 1985;45(2):879-885.
18. Boscato LM, Stuart MC. Heterophilic antibodies: a problem for all immunoassays. *Clin Chem* 1988;34(1):27-33.

Key to Symbols

ISO 15223 Symbols	
	Consult instructions for use
	Manufacturer
Σ	Sufficient for
	Temperature limitation
	Use by/Expiration date
IVD	*In Vitro* Diagnostic Medical Device
LOT	Lot Number
REF	List Number
SN	Serial number

Other Symbols	
ASSAY DILUENT	Assay Diluent
CONJUGATE	Conjugate
CONTAINS: AZIDE	Contains Sodium Azide. Contact with acids liberates very toxic gas.
CONTROL NO.	Control Number
FOR USE WITH	Identifies products to be used together
GTIN	Global Trade Item Number
MICROPARTICLES	Microparticles
PRODUCT OF IRELAND	Product of Ireland
PROTECT FROM LIGHT	Protect from light
REAGENT LOT	Reagent Lot
WARNING: SENSITIZER	Warning: May cause an allergic reaction.

Note for number formatting:

- A space is used as thousands separator (example: 10 000 specimens).
- A period is used to separate the integer part from the fractional part of a number written in decimal form (example: 3.12%).

ARCHITECT and related brand marks are trademarks of Abbott. Other trademarks are the property of their respective owners.

Abbott Ireland
Diagnostics Division
Finisklin Business Park
Sligo
Ireland
+353-71-9171712

Customer Service: Contact your local representative or find country-specific contact information on www.corelaboratory.abbott

Revised May 2020.

©2020 Abbott Laboratories
