# Supplementary material for: Coronavirus Vaccination: Spike Antibody Levels in Health Workers after Six Months—A Cross-Sectional Study
Source: Int J Environ Res Public Health. 2022 Sep 10;19(18):11422. doi: 10.3390/ijerph191811422 (PMC9517660; doi:10.3390/ijerph191811422)
Supplement: Supplementary file 1 [file ijerph-19-11422-s001.zip › ijerph-1878113-supplementary/S2 questionnaire.pdf]

Vaccinations against SARS-CoV-2:

Duration of efficacy of licensed vaccines

vaccines in hospital staff (IDIK)

Dear employee of the Rhein-Maas Klinikum,

Thank you for agreeing to participate in our survey.

The aim of this observational study is to check the S-antibodies present in you after the second vaccination or a vaccination after recovery and to derive from this further measures (e.g. a 3rd vaccination) to ensure protection against infection with SARS-CoV-2 for you as an employee in the clinic and for others.

On the one hand, the vaccination protection is related to existing S-(spike) antibodies, but also still to the cellular immunity of the T-cells, which we do not control in this study.

In addition to the S-antibodies, we check the N-(nucleocapsid) antibodies again as an indication of a previous infection.

The survey takes about 10 minutes on average.

Thank you very much for your participation.

The project team

Contact person at RMK
Dr. Agnes Evenschor-Ascheid, Betriebsärztin
Telefon: +49 2405 62-3151
E-Mail: betriebsarzt@rheinmaasklinikum.de

Contact persons at ZfAM
Lukas Damerau, E-Mail: l.damerau@uke.de,
Dr. Claudia Terschüren, E-Mail: c.terschueren@uke.de
Universitätsklinikum Hamburg-Eppendorf (UKE)
Zentralinstitut für Arbeitsmedizin und Maritime Medizin (ZfAM)
Seewartenstraße 10, Haus 1, 20459 Hamburg

Questionnaire IDIK

1. What ID number did you get when your blood was taken?
(for example 10084)

|_|_|_|_|_|

2. In which year were you born? *(for example 1984)*

|_|_|_|_|

3. What gender are you?

- ○ Male
- ○ Female
- ○ Other

4. What is your profession at the hospital?

- ○ Doctor
- ○ Nurse
- ○ Nursing assistant
- ○ Medical assistant
- ○ Therapist
- ○ Cleaner
- ○ Administrative assistant
- ○ Cleaning/kitchen assistant
- ○ Other profession: ________________________________

5. On which ward / in which area do you mainly work? (Multiple selection possible)

- ○ Emergency room
- ○ Surgical ward
- ○ Non-surgical ward
- ○ Intensive care unit
- ○ Laboratory
- ○ Radiology
- ○ Administration
- ○ Central services (storage, collection and delivery services, technology, gate)
- ○ Housekeeping (cleaning, kitchen)
- ○ Others:____

6. Did you work in a COVID 19 ward in 2021?

This includes, for example, if your other ward was converted for COVID 19 patients.

- ○ Yes
- ○ Exclusively
- ○ No

7. Have you had any contact with a COVID 19 patient while working?

- ○ No (go to question 10)
- ○ Yes, once
- ○ Yes, several times

8. Were you wearing personal protective equipment during the contact?

- ○ No
- ○ Yes

9. Which protective equipment was this? (Multiple selection possible)

- ○ Mask (MNS/FFP2-mask)
- ○ Safety googles/visor (Face Shield)
- ○ Gloves
- ○ Smock

10. Have you had any private contact with people who have / had COVID-19 in the last few months?

- ○ No
- ○ Short, fleeting contact
- ○ Close contact (e.g. family member, partner, flatmate)

11. Have you been in risk areas or a high incidence area, now high risk area, in the last three months?

For example, on holiday or during a business trip.

- ○ No (go to question 13)
- ○ Yes

12. If yes, in which areas were you? (multiple answers possible)

1. ___________________________
2. ___________________________
3. ___________________________

13. Have you had symptoms of illness typical of COVID-19 despite vaccination?

Cough, fever, rhinitis, disturbance of the sense of smell and/or taste

- ○ No (go to question 15)
- ○ Yes

14. If yes, which symptoms were these? (multiple selection possible)

- ○ Rhinitis
- ○ Cough (dry)
- ○ Cough (with sputum)
- ○ Shortness of breath
- ○ Sore throat
- ○ Heart pain (angina pectoris)
- ○ Muscle/limb pain
- ○ Fatigue/exhaustion
- ○ Nausea/vomiting
- ○ Diarrhoea
- ○ Headache
- ○ Loss of taste/odour
- ○ Back pain
- ○ fever, higest temperature |_|_,_|°C, number of days |_|_|_|
- ○ Other symptoms, namely ____

15. Have you been diagnosed with SARS-CoV-2 infection or COVID-19 disease in 2021?

- ○ No (go to question 23)
- ○ Yes, by a positive smear test
- ○ Yes, by positive PCR/antibody test

16. If yes, have you fully recovered from your COVID-19 disease?

- ○ Yes (go to question 19)
- ○ No

17. Which complaints still exist after your COVID-19 disease? (multiple selection possible)

- ○ Exhaustion
- ○ Shortness of breath
- ○ Taste and smell disorders
- ○ Impaired vision
- ○ Dizziness
- ○ Others, namely: ____

18. Have you been given a Long-COVID/Post-COVID diagnosis?

- ○ Yes
- ○ No

19. Did you receive a vaccination after your COVID-19 disease?

- ○ Yes
- ○ No

20. If yes, which vaccine did you receive during this vaccination?

- ○ BioNTech – Comirnaty (mRNA)
- ○ AstraZeneca – Vaxzevria (vector)
- ○ Moderna – Spikevax (mRNA)
- ○ Janssen - COVID-19 Vaccine (vector)

21. In which month in 2021 did you receive this vaccination?

○ January	○ May	○ September
○ February	○ June	○ October
○ March	○ July	○ November
○ April	○ August	○ December

22. Have you noticed any vaccination side effects?

- ○ Yes
- ○ No

For all those who have not had COVID-19:

23. in which month in 2021 did you receive your 1st vaccination?

- ○ January
- ○ February
- ○ March
- ○ April
- ○ May
- ○ June
- ○ July
- ○ August
- ○ September
- ○ October
- ○ November
- ○ December

24. Which vaccine did you receive at your 1st vaccination?

- ○ BioNTech – Comirnaty (mRNA)
- ○ AstraZeneca – Vaxzevria (vector)
- ○ Moderna – Spikevax (mRNA)
- ○ Janssen - COVID-19 Vaccine (vector)

25. What side effects occurred after your 1st vaccination? (multiple answers possible)

- ○ None
- ○ Pain at injection site
- ○ Swelling at injection site
- ○ Tiredness
- ○ Headache
- ○ Pain in limbs
- ○ Joint pain
- ○ Muscle pain
- ○ Chills
- ○ Fever
- ○ Nausea/vomiting
- ○ Other, namely: ____

26. In which month in 2021 did you receive your 2nd vaccination?

- ○ January
- ○ February
- ○ March
- ○ April
- ○ May
- ○ June
- ○ July
- ○ August
- ○ September
- ○ October
- ○ November
- ○ December

27. Which vaccine did you receive for your 2nd vaccination?

- ○ BioNTech – Comirnaty (mRNA)
- ○ AstraZeneca – Vaxzevria (vector)
- ○ Moderna – Spikevax (mRNA)
- ○ Janssen - COVID-19 Vaccine (vector)

28. What side effects occurred after your 2nd vaccination? (multiple answers possible)

- ○ None
- ○ Pain at injection site
- ○ Swelling at injection site
- ○ Tiredness
- ○ Headache
- ○ Pain in limbs
- ○ Joint pain
- ○ Muscle pain
- ○ Chills
- ○ Fever
- ○ Nausea/vomiting
- ○ Other, namely: ____

29. Are you immunosuppressed? Is your immune system weakened by a disease?

- ○ No (questionnaire completed)
- ○ Yes

30. If yes, what weakens your immune system? (multiple selection possible)

- ○ Diabetes mellitus and/or other metabolic diseases
- ○ Cancer
- ○ Inflammatory rheumatic disease (rheumatoid arthritis, ankylosing spondylitis)
- ○ Chronic inflammatory respiratory disease (COPD, asthma)
- ○ chronic heart, liver or kidney disease
- ○ Chronic inflammatory bowel disease (Crohn's disease, ulcerative colitis)
- ○ Chronic nerve disease (MS, epilepsy, cerebral palsy)
- ○ HIV infection/AIDS
- ○ Taking immunosuppressants
- ○ Other, namely ____

You have reached the end of the questionnaire.

Thank you very much for your participation!

Please hand in the questionnaire to Dr. Evenschor-Ascheid at the same time as you take your blood sample (same ID number).
